# Prevalence of Agitation and Its Associated Factors in Adult Intensive Care Unit: A Systematic Review and Meta‐Analysis

**DOI:** 10.1111/nicc.70296

**Published:** 2025-12-11

**Authors:** Temesgen Birlie Asmare, Getachew Mekete Diress, Negesse Zurbachew Gobezie, Abere Gebru Abuhay, Walelign Asime Afework, Gezahagn Demsu Gedefaw, Asnake Tadesse Abate, Biruk Demissie, Tsehayu Melak Siyoum, Begizew Yimenu Mekuriaw, Habtie Bantider Wubet

**Affiliations:** ^1^ Department of Anesthesia, School of Medicine, College of Health Sciences Debre Tabor University Debre Tabor Ethiopia; ^2^ Department of Pediatrics and Child Health Nursing, College of Health Sciences Debre Tabor University Debre Tabor Ethiopia; ^3^ Department of Adult Health Nursing, College of Health Sciences Debre Tabor University Debre Tabor Ethiopia; ^4^ Department of Neonatal Health Nursing, School of Nursing, College of Medicine and Health Sciences, and Specialized Hospital University of Gondar Gondar Ethiopia; ^5^ Department of Environmental Health, College of Health Science Debre Tabor University Debre Tabor Ethiopia; ^6^ Department of Neonatal Health Nursing, School of Nursing, College of Medicine and Health Sciences University of Gondar Gondar Ethiopia; ^7^ Department of Midwifery, College of Health Sciences Debre Tabor University Debre Tabor Ethiopia

**Keywords:** agitation, associated factors, critically ill patients, incidence, intensive care unit, prevalence

## Abstract

**Background:**

Agitation is a common and clinically significant phenomenon among patients admitted to intensive care units (ICUs), particularly those receiving mechanical ventilation or experiencing critical illness. Previous studies have shown inconsistent results regarding the prevalence and predictors of agitation in intensive care units.

**Aim:**

To assess the pooled prevalence and associated factors of agitation among adults in the intensive care unit.

**Study Design:**

A systematic review and meta‐analysis was conducted. The review protocol has been registered in the Prospero database under registration number CRD420251022240, following PRISMA guidelines.

**Results:**

This systematic review and meta‐analysis included 10 studies. The pooled prevalence of agitation in the intensive care unit was 55.65% (95% CI: 40.07, 71.24). The pooled analysis revealed a significant association between hyperthermia (≥ 37.5°C) and the prevalence of agitation in adult patients in the intensive care unit. The adjusted odds ratio (AOR) for hyperthermia was 3.24 (95% CI: 1.51–4.91, *p* < 0.0002).

**Conclusion:**

This meta‐analysis highlights the significant burden of agitation among adult patients in intensive care units, revealing that over half of critically ill individuals experience agitation during their ICU stay. Among the various contributing factors examined, elevated body temperature emerged as the only one with a statistically significant association with agitation.

**Relevance to Clinical Practice:**

This study underscores the importance of vigilant temperature monitoring and timely management of fever in ICU patients. Hyperthermia was found to be associated with a higher likelihood of agitation, although a direct causal relationship cannot be established from the available data. Maintaining normothermia remains a reasonable clinical goal that may contribute to overall patient stability. Implementing structured temperature control protocols as part of routine ICU care could help reduce agitation‐related complications such as unplanned extubation, increased sedation needs and prolonged ICU stays, thereby supporting improved patient safety and outcomes.

## Impact Statements

- What is known about this topic?
  ○ Agitation is a frequent complication among critically ill ICU patients. It is linked to adverse outcomes such as unplanned extubation and increased sedation needs.
  ○ Previous studies have reported inconsistent findings regarding the prevalence and predictors of agitation in the ICU.
- What this paper adds?
  ○ Provides pooled evidence showing that over half of ICU patients experience agitation (55.65%).
  ○ Identifies hyperthermia (≥ 37.5°C) as a strong and significant predictor of agitation, tripling the risk.
  ○ Highlights fever control as a practical, modifiable target for early prevention of agitation in ICU clinical practice.

**Abbreviations**
AJOL: African Journal Online
AMSTAR: Assessing the methodological quality of systematic reviews
AOR: Adjusted Odds Ratio
CI: Confidence Interval
ICU: Intensive Care Unit
NOS: Newcastle–Ottawa Scale
PRISMA: Preferred Reporting Items for Systematic Review and Meta‐analysis
RASS: Richmond Agitation‐Sedation Scale
STATA: Statistical Software Package

## 1 Introduction

Agitation is a prevalent and clinically significant phenomenon among patients admitted to intensive care units (ICUs), particularly among adults receiving mechanical ventilation or experiencing critical illness [1]. It is typically characterised by restlessness, excessive motor activity, verbal or physical aggression, and non‐purposeful movement, which can disrupt medical care and pose a danger to both patients and healthcare staff [1, 2, 3]. The aetiology of agitation in ICU patients is multifactorial. Contributing factors include sedation practices, benzodiazepine use, sleep deprivation, metabolic disturbances, mechanical ventilation and environmental influences such as noise and light [4, 5]. Agitation in the ICU is often associated with underlying delirium, pain, hypoxia, drug withdrawal or the administration of certain medications; however, it can also occur independently of these factors [6].

The prevalence of agitation varies widely across studies and regions, with reported rates ranging from 17.5% to 89% among ICU patients, depending on the population studied, definitions used and assessment tools applied [7, 8]. The lack of a universally accepted definition or diagnostic criteria for agitation contributes to the variability in reported prevalence rates [9].

Agitation can lead to unfavourable outcomes such as prolonged stays in the intensive care unit (ICU), prolonged mechanical ventilation, increased frequency of nosocomial infections, higher unplanned extubations, removal of the central venous catheter and higher frequency of surgical re‐intervention due to anastomotic leaks [3, 10]. Furthermore, agitation can exacerbate other undesirable complications such as pain, anxiety and delirium, which contribute to further physiological derangements and damage to the patient [11].

Despite increasing recognition of its clinical significance, the burden of agitation in ICUs remains under‐characterised. Most existing studies have been conducted in high‐income countries, with limited data available from low‐ and middle‐income nations, where differences in sedation practices, ICU staffing and resource availability may influence the prevalence and management of agitation [12].

Managing agitation in the intensive care unit (ICU) requires a multimodal approach that integrates both non‐pharmacological and pharmacological interventions [1, 13, 14]. Non‐pharmacological strategies—such as reorientation, ensuring adequate sleep, early mobilisation and minimising environmental stressors—are first‐line interventions designed to address underlying causes [1]. Pharmacological treatments—primarily antipsychotics (e.g., haloperidol) and sedatives (e.g., dexmedetomidine, benzodiazepines)—are employed when non‐drug methods prove ineffective or when agitation poses safety risks. However, these medications come with potential risks, including over sedation, prolonged ICU stays and delirium [15, 16, 17]. Challenges in management include difficulties in accurate assessment due to overlapping symptoms with delirium, variability in patient responses to medications, limited evidence supporting pharmacologic agents and ethical concerns regarding the overuse of sedation [9].

## 2 Background/Justification for Review

Previous studies have reported inconsistent results regarding the prevalence of agitation in intensive care units, ranging from 17.5% [7] to 89% [8]. To the authors' knowledge, no systematic review or meta‐analysis has thoroughly examined the prevalence and risk factors for agitation among adult ICU patients. This underscores the urgent need for a comprehensive synthesis of existing evidence. A systematic review and meta‐analysis can provide valuable insights by aggregating data from diverse studies, identifying patterns, assessing the quality of evidence and quantifying the burden of agitation across various settings. This study aims to conduct the first systematic review and meta‐analysis to determine the prevalence of agitation and identify associated factors in adult ICU patients. By addressing these gaps and establishing a robust evidence base, the study seeks to make a significant contribution to clinical practice, research and policymaking in critical care medicine.

## 3 Aim

To determine the pooled prevalence of agitation and identify its associated factors among adult patients admitted to intensive care units (ICUs).

## 4 Specific Objectives

- To estimate the pooled prevalence of agitation among adult ICU patients.
- To identify and analyse factors associated with agitation in critically ill adult ICU patients.

## 5 Focused Research Question

PEO (Population: An adult patient has been admitted to the intensive care unit; Exposure: associated factors; Outcome: prevalence of agitation).

In adult patients admitted to the intensive care unit (ICU), what is the prevalence of agitation and what factors are associated with its occurrence?

## 6 Methods

### 6.1 Reporting and Registration Protocol

This systematic review and meta‐analysis were reported following the Preferred Reporting Items for Systematic Reviews and Meta‐Analyses (PRISMA) checklist. This checklist provides a standardised framework to ensure transparency and completeness in reporting systematic reviews and meta‐analyses [18]. The review protocol has been registered in the Prospero database under the registration number (CRD420251022240).

### 6.2 Eligibility Criteria

The review includes observational studies, original articles and research involving adults that examined the prevalence of agitation, associated factors or both in the intensive care unit, all published in English. However, it excludes reviews, commentaries, editorials, case reports, qualitative studies, non‐human or paediatric studies, as well as duplicates or studies with overlapping populations.

### 6.3 Literature Search Strategy

We conducted a systematic review and meta‐analysis of published articles to estimate the pooled prevalence of agitation and its associated factors in intensive care units. Our systematic search included the following electronic databases: PubMed/MEDLINE, Science Direct, Cochrane Library and Web of Science. We also searched Google Scholar to identify grey literature (Supplementary 1). Our search strategy incorporated key terms such as adult patients, agitation, associated factors, critically ill patients, incidence or prevalence, and intensive care unit, along with their MeSH terms. Boolean operators and word truncation (*) were used to manage spelling variations during the search. Both free‐text and Medical Subject Headings (MeSH) terms were employed while searching the literature in online databases. The search strategy for the PubMed database was as follows: ((((((((((((Incidence[Title/Abstract]) OR (‘epidemiology’[Subheading])) OR (‘incidence’[MeSH Terms])) OR (Prevalence[Title/Abstract])) OR (Magnitude[Title/Abstract])) OR (Proportion[Title/Abstract])) OR (Occurrence[Title/Abstract])) OR (Frequency[Title/Abstract])) OR (Emergence[Title/Abstract])) AND ((((((Agitation[Title/Abstract]) OR (Hyperactivity[Title/Abstract])) OR (Aggression[Title/Abstract])) OR (‘Psychomotor agitation’[Title/Abstract])) OR (Combativeness[Title/Abstract])) OR (Delirium[Title/Abstract]))) AND (((((((‘Associated Factors’[Title/Abstract]) OR (Determinants[Title/Abstract])) OR (‘Epidemiologic factors’[Title/Abstract])) OR (Predictors[Title/Abstract])) OR (‘Risk factors’[Title/Abstract])) OR (‘Contributing Factors’[Title/Abstract])) OR (Correlates[Title/Abstract]))) AND (((((((Adult[Title/Abstract]) OR (‘adult’[MeSH Terms])) OR (‘Post‐adolescent’[Title/Abstract])) OR (‘Young adult’[Title/Abstract])) OR (‘Middle‐aged’[Title/Abstract])) OR (Elderly[Title/Abstract])) OR (‘Adult patients’[Title/Abstract]))) AND ((((‘Intensive Care Unit’[Title/Abstract]) OR (‘Intensive care’[Title/Abstract])) OR (‘Critical care’[Title/Abstract])) OR (ICU[Title/Abstract])) (Supplementary 1). The search was repeated to verify the consistency of the search terms and results. The search was conducted from 10 March 2025, to 7 May 2025. Two authors (TBA and GMD) independently performed the article searches, while the third investigator (HBW) resolved any inconsistencies. All included studies were published between 2000 [19] and 2025 [3].

### 6.4 Study Selection

All retrieved studies were exported to EndNote version 21, where duplicates were removed. Three independent reviewers (TBA, GMD and HBW) then conducted a thorough screening process to determine each study's eligibility. They first screened the titles and abstracts to assess relevance to the inclusion criteria. Studies that met the initial criteria underwent a full article review to confirm their eligibility. Any discrepancies between the reviewers regarding study eligibility or data extraction were resolved through discussion.

### 6.5 Data Extraction

Studies that met the inclusion criteria were screened, and data were extracted by reviewers (TBA, GMD, HBW, GDG and BD) using a Microsoft Excel spreadsheet. The following information was systematically collected from each study: the first author's name, year of publication, study area, study design, sample size, prevalence of agitation and factors associated with agitation.

### 6.6 Data Synthesis and Analysis

All statistical analyses were conducted using STATA version 17. The overall prevalence of agitation and its associated factors were calculated using a weighted inverse‐variance random‐effects model [20]. Publication bias was assessed by examining the symmetry of the funnel plot. Significant bias was identified through Egger's test, which yielded a *p* < 0.05 [21]. The percentage of total variation across studies due to heterogeneity was assessed using *I* ^2^ statistics. *I* ^2^ values of 25%, 50% and 75% indicate low, moderate and high levels of heterogeneity, respectively [22]. Significant heterogeneity was defined as when the *p*‐value of the *I* ^2^ statistic < 0.05. Sensitivity analysis was done to determine the impact of a particular study on the entire meta‐analysis. We identified the factors significantly associated with the incidence of agitation in the intensive care unit using STATA version 17. This meta‐analysis utilised a random‐effects model to account for variability between studies [23, 24]. Additionally, we conducted subgroup analysis, meta‐regression and the trim‐and‐fill test. The results were presented in text, tables and various plots, including measures of effect and a 95% confidence interval.

A separate meta‐analysis was conducted to determine pooled estimates of the factors associated with the prevalence of agitation in adult intensive care units. Variables were included in the quantitative synthesis only if they were assessed in at least two independent studies with comparable definitions and outcome measures. For each variable, we extracted either crude or adjusted odds ratios (ORs) along with their corresponding 95% confidence intervals (CIs), prioritising adjusted odds ratios. When ORs were not directly reported, we calculated them from the available 2 × 2 contingency tables. Extracted data were independently coded and entered into a standardised spreadsheet by two reviewers to ensure accuracy and consistency. Discrepancies were resolved through consensus or consultation with a third reviewer. Statistical analyses were performed using STATA version 17. Heterogeneity across studies was assessed using Cochran's Q test (with *p* < 0.10 indicating significant heterogeneity) and quantified using the *I* ^2^ statistic (values of 25%, 50% and 75% representing low, moderate and high heterogeneity, respectively). A random‐effects model (DerSimonian and Laird method) was applied to generate pooled estimates, accounting for between‐study variability.

### 6.7 Outcome Measures of Interest

The primary outcome of interest was the prevalence of agitation in adult intensive care units, while the secondary outcome focused on the factors influencing this prevalence in those settings.

### 6.8 Methods for Assessing the Risk of Bias in Studies

To identify potential biases in studies and limitations in data analysis and result reporting, we utilised the STROBE statement. This statement offers recommendations aimed at improving the presentation of results from observational studies [25]. Additionally, to ensure the quality of each study, we used a modified Newcastle–Ottawa Scale (NOS) appraisal assessment tool established for cross‐sectional, case–control and cohort studies [26]. Each author independently assessed the quality of the studies using the Newcastle–Ottawa Scale. This scale evaluates several criteria, including sample representativeness, adequacy, measurement tools for exposure or risk factors, response rates, comparability of outcome groups, control of confounding variables, outcome evaluation and statistical tests, with a maximum possible score of 10 points. A total score greater than 5 out of 10 for each study indicated a low risk of bias, allowing for its inclusion in the review. All included studies confirmed a low risk of bias, scoring above 6 out of 10.

## 7 Results

### 7.1 Search Results

**FIGURE 1:**
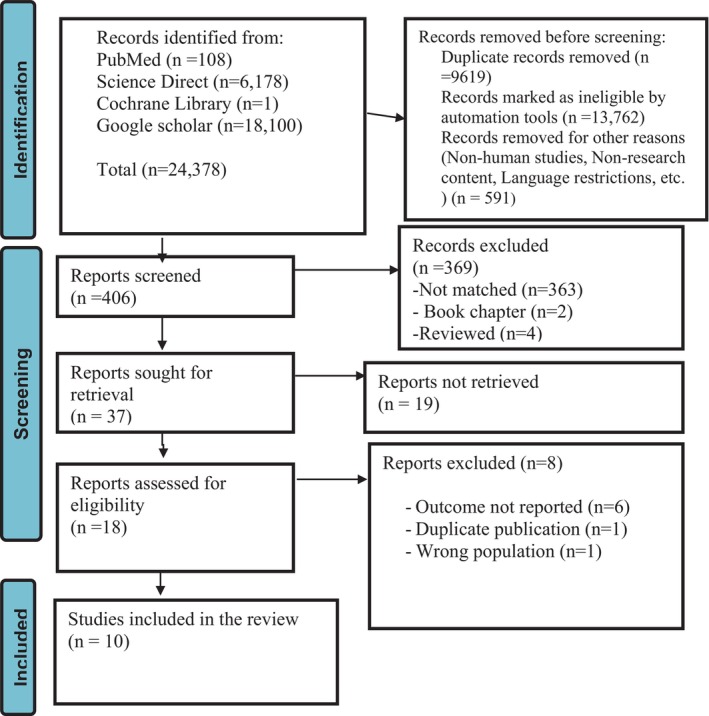
PRISMA flow diagram showing search databases and results.

The article selection and screening process presented in the PRISMA flow chart (Figure 1) [18]. Finally, 10 studies were relevant to determining the prevalence of agitation and associated factors among adults in the intensive care unit.

### 7.2 Characteristics of the Included Studies

All studies [3, 7, 8, 10, 19, 27, 28, 29, 30, 31] were prospective observational studies. Based on geographical location, studies were one from Ethiopia [3], one from Qatar [30], one from Turkey [8], one from Greece [29], one from France [10], one from Brazil [27], three from the USA [7, 19, 28] and one from Canada [31]. A total of 1306 study participants were included from all studies, with nearly two‐thirds (31.4% or 410 participants) coming from the USA. The smallest sample size was 30 [31], while the largest was 253 [3] (Table 1).

**TABLE 1:** Characteristics of the included studies to assess the pooled prevalence and associated factors of agitation among adult patients admitted in the intensive care unit.

S/no	Author (year)	Country	ICU discipline	Assessment tool	Agitated (*n* (%))	Non‐agitated (*n* (%))	Risk factors assessed
1	Jaber et al. (2005) [10]	France	Mixed ICU	Modified RAMSAY	95 (52)	87 (42)	‐ Psychoactive drug use ‐ History of alcohol abuse ‐ Dysnatraemia ‐ Fever ‐ Use of sedatives in the ICU ‐ Sepsis
2	Burk et al. (2014) [28]	USA	Mixed ICU	RASS ≥ 1	118 (59)	82 (41)	‐ Past medical history of psychiatric diagnosis ‐ Height ‐ SOFA score ‐ P/F < 200 mmHg ‐Serum pH ‐ Per cent of hours using restraints ‐ Per cent of hours using mechanical ventilation ‐Pain ‐ Presence of genitourinary catheters
3	Kiekkas et al. (2010) [29]	Greece	Mixed ICU	Riker ASS	75 (46.6)	86 (53.4)	‐ Fever
4	Asmare et al.(2025) [3]	Ethiopia	Mixed ICU	RASS ≥ 1	221 (87.35)	32 (12.65)	‐ Anxiety ‐ Delirium ‐ Pain ‐ Hyperthermia ‐ Hyponatraemia ‐ The use of restraints
5	Mahmood et al. (2018) [30]	Qatar	Trauma ICU	RAMSAY	46 (45)	56 (55)	‐ Use of propofol alone ‐ Subarachnoid Haemorrhage ‐ ICP catheter insertion for severe head injury
6	Almeida et al. (2016) [27]	Brazil	Mixed ICU	RASS ≥ 2	36 (31.8)	77 (68.2)	‐ Delirium ‐ Moderate or severe pain ‐ Mechanical ventilation ‐ Smoking habits ‐ Hyperlactataemia (protective)
7	Bahar et al. (2023) [8]	Turkey	Mixed ICU	RASS ≥ 1	49 (89)	6 (11)	—
8	Arroyo‐Novoa et al. (2019) [7]	USA	Mixed ICU	RASS ≥ 1	14 (17.5)	66 (83.5)	—
9	Williamson et al. (2020) [31]	Canada	Trauma ICU	RASS ≥ 2	17 (56.7)	13 (43.3)	—
10	Fraser et al. (2000) [19]	USA	Mixed ICU	SAS	92 (70.8)	38 (29.2)	—

*Note:* ‘—’ means, indicates studies that did not investigate associated factors.

Abbreviations: ICU, intensive care unit; *n* (%), frequency (percentage); RASS, Richmond Agitation–Sedation Scale; Riker ASS, Riker Agitation–Sedation Scale; SAS, Sedation Agitation Scale; SOFA score, sequential organ failure assessment score.

### 7.3 Quality Appraisal of the Included Studies

The quality of the included studies was assessed by three independent authors (TBA, GMD and HBW), who also provided scores for the validity of the results. Each study was evaluated using the modified Newcastle–Ottawa Scale (NOS) quality rating standards. As a result, two studies [3, 10] scored 10 out of 10 questions, three studies [8, 29, 30] scored nine, four studies [19, 27, 28, 31] scored eight and one study [7] scored 7 out of 10. A total score greater than 5 out of 10 indicated a low risk of bias for each study. Following a thorough quality evaluation, all included studies demonstrated a high level of methodological quality and dependability. Each study received a score between 7 and 10 out of a possible 10 points, confirming that the quality of all included studies was high.

### 7.4 Meta‐Analysis

#### 7.4.1 Prevalence of Agitation

**FIGURE 2:**
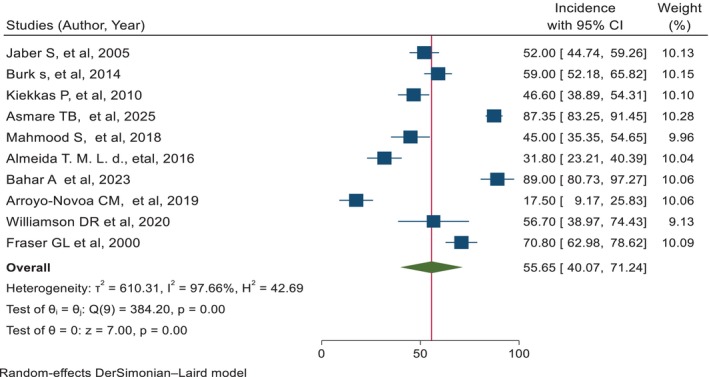
The pooled prevalence of agitation among adult patients in the intensive care unit, along with a 95% confidence interval.

Finally, the meta‐analysis contained 10 studies [3, 7, 8, 10, 19, 27, 28, 29, 30, 31]. The prevalence of agitation in adult intensive care units varies from 17.50% [7] to 89.00% [8], while the overall pooled prevalence of agitation was 55.65% (95% CI: 40.07, 71.24); *I* ^2^ = 97.66%; *p* < 0.001 (Figure 2).

#### 7.4.2 Publication Bias

**FIGURE 3:**
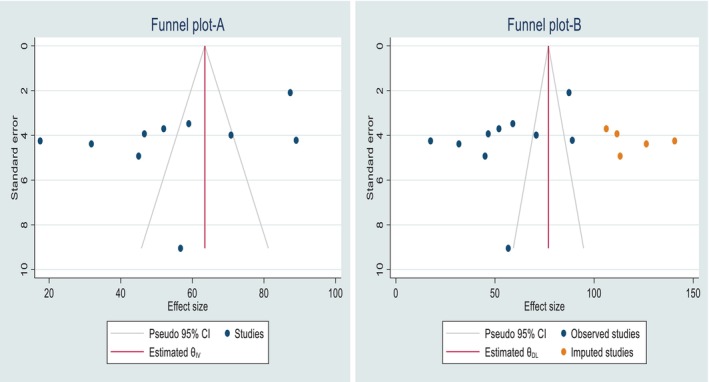
Funnel plot illustrating the results before adjustment (A) and after adjustment (B) using trim‐and‐fill analysis to assess publication bias related to the prevalence of agitation among adult ICU patients.

The asymmetric distribution of studies in the funnel plot suggests potential publication bias (Figure 3A). However, the *p*‐value from Egger's regression test (*p* = 0.4925) indicates no statistically significant evidence of such bias. To address the asymmetry in the funnel plot, we conducted a trim‐and‐fill analysis (Figure 3B).

#### 7.4.3 Investigation of Heterogeneity

The *I* ^2^ statistic from the forest plot indicates significant heterogeneity among the included studies (*I* ^2^ = 97.66%, *p* < 0.001) (Figure 2). Consequently, sensitivity and subgroup analyses were conducted to minimise this heterogeneity.

#### 7.4.4 Sensitivity Analysis

**FIGURE 4:**
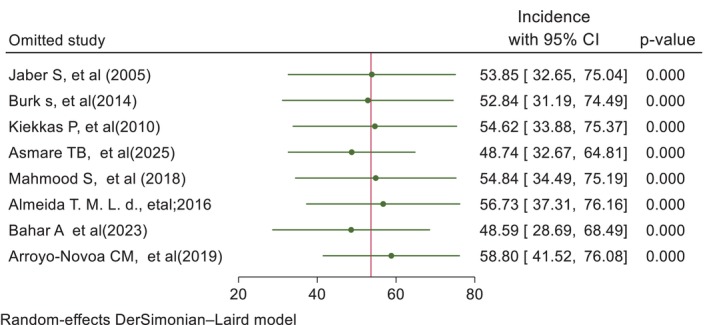
Forest plot illustrating the sensitivity analysis of the prevalence of agitation among adult ICU patients.

To assess the robustness of the pooled prevalence estimate, a sensitivity analysis was conducted by sequentially excluding each study from the meta‐analysis. The results indicated that the overall pooled effect size was stable and ranged from 55.65% (95% CI: 40.07% to 71.24%), with no single study significantly altering the overall effect. For example, exclusion of individual studies such as Jaber et al. [10] or Bahar et al. [8] resulted in minimal changes to the overall estimate (Figure 4). This consistency suggests that the findings are not unduly influenced by any one study. Furthermore, when the random‐effects model was replaced with a fixed‐effects model, the pooled prevalence was slightly increased (63.503; 95% CI: 61.21–65.80), but the direction and statistical significance remained consistent. These findings suggest that the results are robust and not sensitive to individual studies or analytic models.

#### 7.4.5 SubGroup Analysis by the Sample Size

Subgroup analysis was conducted based on the sample sizes of the included studies, categorising them into two groups: those with a sample size of ≤ 131 participants and those with a sample size of > 131 participants. This classification aimed to investigate whether study size influences the prevalence of agitation.

**FIGURE 5:**
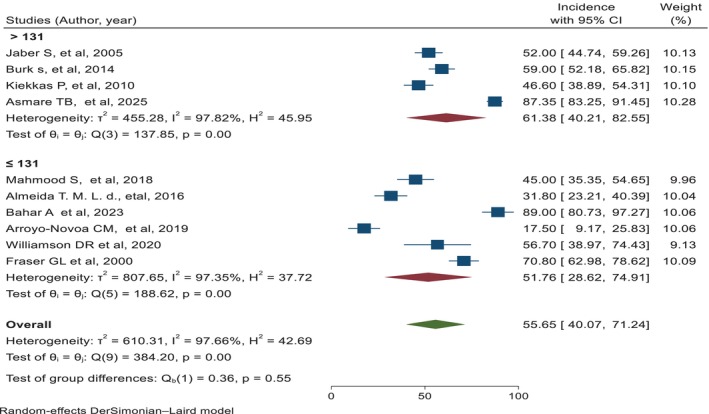
Forest plot showing the subgroup analysis of the prevalence of agitation, with a 95% confidence interval, among adult ICU patients.

The pooled prevalence for studies with smaller sample sizes (≤ 131) was 51.76% (95% CI: 28.62, 74.91), while for studies with larger sample sizes (> 131), the pooled prevalence was 61.38% (95% CI: 40.21, 82.55) (Figure 5). The test for subgroup differences revealed no statistically significant difference between the two groups (Qb(1) = 0.36, *p* = 0.55), indicating that sample size did not significantly moderate the overall prevalence of agitation. This finding suggests that the results of the meta‐analysis are consistent regardless of study size.

#### 7.4.6 Factors Associated With Prevalence of Agitation

Five factors that were found to be statistically significant in at least two primary studies were included in the meta‐analysis to calculate the pooled Adjusted Odds Ratios (AORs). These factors were: pain, hyponatremia, delirium, use of restraint and hyperthermia (> 37.5°C).

The pooled analysis revealed that only hyperthermia (> 37.5°C) was significantly associated with the prevalence of agitation among adult patients in the intensive care unit. The pooled AOR for hyperthermia (> 37.5°C) was 3.24 (95% CI: 1.51–4.91, *p* < 0.0002), indicating that individuals who had hyperthermia had more than three times the odds of experiencing agitation compared to those who had no hyperthermia. (Table 2).

**TABLE 2:** Pooled adjusted odds ratios (AORs) for factors associated with agitation among ICU patients.

S. No	Factors	No. of included studies	Pooled AOR (95% CI)	*p*	Statistical significant
1	Hyperthermia (> 37.5°C)	3	3.24 (1.51–4.91)	< 0.0002	Yes
2	Pain	3	1.66 (−0.25, 3.57)	0.0891	No
3	Hyponatraemia	2	4.216 (−0.91, 9.34)	0.1068	No
4	Delirium	2	11.11 (−9.03, 31.25)	0.2795	No
5	Use of restraint	2	1.26 (−0.44, 2.96)	0.1458	No

Abbreviation: AOR, adjusted odds ratio.

## 8 Discussion

This meta‐analysis synthesised evidence from 10 observational studies to estimate the prevalence of agitation among adult ICU patients and identify its associated clinical risk factors. The pooled prevalence of agitation was 55.65% (95% CI: 40.07–71.24), indicating that more than half of critically ill adults experience agitation during their ICU stay. Reported prevalence varied widely—from 17.5% to 89%—due to differences in patient characteristics, sedation protocols, assessment tools and ICU management strategies across different settings. Despite substantial heterogeneity (*I* ^2^ = 97.66%, *p* < 0.001), sensitivity analysis confirmed the robustness of this pooled estimate, with no single study exerting undue influence. Furthermore, both fixed‐ and random‐effects models produced consistent findings, further supporting the reliability of the results.

A subgroup analysis based on study sample size (≤ 131 vs. > 131 participants) found no significant difference in the prevalence of agitation between small and large studies, indicating that study size did not meaningfully impact the outcome. Although visual inspection of the funnel plot suggested potential publication bias, Egger's test (*p* = 0.4925) and trim‐and‐fill analysis indicated that this bias was not statistically significant or influential, thereby supporting the validity and credibility of the results.

In addition to estimating prevalence, this meta‐analysis examined five clinical factors previously associated with ICU agitation: pain, hyponatremia, delirium, use of restraint and hyperthermia. Among these factors, only hyperthermia (> 37.5°C) showed a statistically significant association with agitation, with a pooled AOR of 3.24 (95% CI: 1.51–4.91, *p* < 0.0002). This indicates that ICU patients with elevated body temperature have a more than threefold higher likelihood of being associated with agitation compared to normothermic patients.

The strong association between hyperthermia and agitation is biologically plausible. Elevated body temperature serves as a systemic inflammatory trigger, stimulating the release of pro‐inflammatory cytokines such as IL‐1β, IL‐6 and TNF‐α. These cytokines can disrupt the blood–brain barrier and promote neuroinflammation, factors that are strongly implicated in the development of delirium and agitation [32, 33]. Additionally, fever increases the body's metabolic rate and cerebral oxygen demand. In critically ill patients, this can lead to relative cerebral hypoxia or metabolic distress, impairing consciousness and potentially triggering agitation [34].

In contrast, the other four factors—pain, hyponatremia, delirium and restraint use—did not show statistically significant associations in the pooled analysis, even though individual studies suggested possible links. These discrepancies may stem from clinical and methodological heterogeneity, variations in assessment tools and differences in ICU practices. For instance, delirium and agitation frequently co‐occur, yet their diagnostic overlap and varying definitions likely contribute to inconsistent associations [1, 35]. Similarly, restraint use often follows agitation as a clinical response, but it may also provoke agitation, complicating causal interpretations [36, 37]. Pain, particularly in non‐verbal patients, may be under‐recognised or misclassified [38], while hyponatremia may only produce cognitive effects at extreme levels [39].

These findings collectively underscore hyperthermia as a potentially modifiable factor associated with agitation and highlight the importance of systematic temperature monitoring and management protocols in ICU settings. The high pooled prevalence of agitation emphasises the need for routine assessments, early recognition and preventive strategies tailored to critically ill patients. Nonetheless, this association should be interpreted with caution given the multifactorial nature of agitation, which is influenced by factors such as illness severity, sedative use, metabolic disturbances and environmental stimuli. Moreover, the limited number of studies contributing to this and other predictor analyses may affect the robustness and generalisability of the findings. Therefore, temperature management should be regarded as one component of a comprehensive, multifaceted approach to reducing agitation and improving patient outcomes in the ICU.

## 9 Strengths and Limitations

This meta‐analysis has several strengths. It is the first to comprehensively report both the pooled prevalence of ICU agitation and its associated clinical factors using rigorous meta‐analytic methods. The inclusion of sensitivity analyses, subgroup comparisons and multiple statistical models enhances the reliability of the findings. Additionally, the study employed funnel plots, Egger's test and trim‐and‐fill procedures to explore and adjust for publication bias, further supporting the validity of the results.

Several limitations must be acknowledged in this meta‐analysis. First, there was substantial heterogeneity among the included studies (*I* ^2^ = 97.66%), likely due to variations in definitions of agitation, measurement tools, patient characteristics and ICU protocols. Although Egger's test did not indicate statistical evidence of publication bias, the small number of studies limits the power to reliably detect such bias. Furthermore, the inclusion of only 10 studies may restrict generalisability, and the findings are based on observational data, which precludes causal inference. The relatively small number of studies included for each factor may also limit the statistical power and generalisability of the results. Additionally, considerable heterogeneity in study design, patient populations and definitions of agitation and predictor variables could influence pooled estimates. The reliance on observational studies further restricts causal inference, as factors such as restraint use may both cause and result from agitation. Moreover, variations in measurement tools and potential residual confounding from unmeasured variables may have affected the accuracy of the pooled AORs. Lastly, publication bias and language restrictions may have led to the exclusion of relevant studies, potentially impacting the comprehensiveness of the results.

## 10 Conclusion and Recommendations

This meta‐analysis highlights the significant burden of agitation among adult patients in intensive care units, revealing that over half of critically ill individuals experience agitation during their ICU stay. Although the included studies showed considerable variability, indicated by a high degree of heterogeneity, further analyses confirmed that the overall results were consistent and reliable, regardless of study size or methodology. Among the various contributing factors examined, elevated body temperature emerged as the only one with a statistically significant association with agitation. Patients with fever had more than threefold higher odds of being associated with agitation compared to those with normal body temperature, highlighting hyperthermia as an observed clinical correlate rather than a confirmed causal factor.

Based on these findings, routine monitoring and appropriate management of hyperthermia should remain a key aspect of ICU practice, given its observed association with increased agitation among critically ill patients. Standardised agitation assessment tools, particularly the Richmond Agitation‐Sedation Scale (RASS), should be consistently used to improve early detection and intervention. Moreover, ICU protocols should be reviewed to incorporate targeted interventions for febrile patients, including appropriate antipyretic use and non‐pharmacological comfort measures, which may help reduce the burden of agitation and improve patient outcomes. Future researchers are encouraged to include a larger number of studies and to design well‐powered, multicentre investigations that use standardised definitions of agitation and predictor variables, thereby strengthening the evidence base, improving the robustness of conclusions and enhancing the comparability and generalisability of findings in critical care. Employing uniform diagnostic criteria and validated assessment tools across diverse ICU populations will reduce heterogeneity and improve the accuracy of pooled estimates.

## Author Contributions

G.M.D., N.Z.G. and T.B.A.: conceptualization, methodology, software, data curation, writing – original draft preparation, visualization, investigation, supervision, validation, and writing – reviewing and editing. H.B.W., W.A.A., A.G.A., D.G.D.: Methodology, software, visualization, investigation, supervision, validation and editing. A.T.A., T.M.S., B.Y.M., G.D.G. and B.D.: methodology, software, data curation, writing – original draft preparation, visualization, and writing – reviewing and editing. All authors reviewed the manuscript and agreed to publication.

## Funding

The authors have nothing to report.

## Ethics Statement

The authors have nothing to report.

## Consent

The authors have nothing to report.

## Conflicts of Interest

The authors declare no conflicts of interest.

## Supporting information


**Data S1:** Supplementary Information.

## Data Availability

Data sharing not applicable to this article as no datasets were generated or analysed during the current study.
