# Supplementary material for: Prevalence of Agitation and Its Associated Factors in Adult Intensive Care Unit: A Systematic Review and Meta‐Analysis
Source: Nurs Crit Care. 2025 Dec 11;31(1):e70296. doi: 10.1111/nicc.70296 (PMC12697384; doi:10.1111/nicc.70296)
Supplement: Supplementary file 1 — Data S1: Supplementary Information. [file NICC-31-0-s001.doc]

|  | Concept 1 | Concept 2 | Concept 3 | Concept 4 | Concept 5 |
| --- | --- | --- | --- | --- | --- |
| Free text word | Incidence | Agitation | “Associated Factors” | Adult | “Intensive Care Unit” |
| Mesh | "epidemiology"[Subheading] OR "incidence"[MeSH Terms] | - | - | "adult"[MeSH Terms] | - |
| Related Terms | Prevalence  Magnitude  Proportion  Occurrence  Frequency  Emergence | Hyperactivity  Aggression  “Psychomotor agitation”  Combativeness | Determinants  “Epidemiologic factors”  Predictors  “Risk factors”  “Contributing Factors”  Correlates | “Post-adolescent”  “Young adult”  “Middle-aged”  Elderly  “Adult patients” | “Intensive care”  “Critical care”  ICU |

**Prevalence of Agitation and Its Associated Factors in Adult Intensive Care Unit: A Systematic Review and Meta-analysis.**

**Pub Med=108**

| Search in each key words and Mesh terms | **Pub med/Medline database** |
| --- | --- |
| **#1** | ((((((((Incidence[Title/Abstract]) OR ("epidemiology"[Subheading])) OR ("incidence"[MeSH Terms])) OR (Prevalence[Title/Abstract])) OR (Magnitude[Title/Abstract])) OR (Proportion[Title/Abstract])) OR (Occurrence[Title/Abstract])) OR (Frequency[Title/Abstract])) OR (Emergence[Title/Abstract]) |
| **#2** | (((((Agitation[Title/Abstract]) OR (Hyperactivity[Title/Abstract])) OR (Aggression[Title/Abstract])) OR ("Psychomotor agitation"[Title/Abstract])) OR (Combativeness[Title/Abstract]) |
| **#3** | (((((("Associated Factors"[Title/Abstract]) OR (Determinants[Title/Abstract])) OR ("Epidemiologic factors"[Title/Abstract])) OR (Predictors[Title/Abstract])) OR ("Risk factors"[Title/Abstract])) OR ("Contributing Factors"[Title/Abstract])) OR (Correlates[Title/Abstract]) |
| **#4** | ((((((Adult[Title/Abstract]) OR ("adult"[MeSH Terms])) OR ("Post-adolescent"[Title/Abstract])) OR ("Young adult"[Title/Abstract])) OR ("Middle-aged"[Title/Abstract])) OR (Elderly[Title/Abstract])) OR ("Adult patients"[Title/Abstract]) |
| **#5** | ((("Intensive Care Unit"[Title/Abstract]) OR ("Intensive care"[Title/Abstract])) OR ("Critical care"[Title/Abstract])) OR (ICU[Title/Abstract]) |
| **Final** | ((((((((((((Incidence[Title/Abstract]) OR ("epidemiology"[Subheading])) OR ("incidence"[MeSH Terms])) OR (Prevalence[Title/Abstract])) OR (Magnitude[Title/Abstract])) OR (Proportion[Title/Abstract])) OR (Occurrence[Title/Abstract])) OR (Frequency[Title/Abstract])) OR (Emergence[Title/Abstract])) AND ((((((Agitation[Title/Abstract]) OR (Hyperactivity[Title/Abstract])) OR (Aggression[Title/Abstract])) OR ("Psychomotor agitation"[Title/Abstract])) OR (Combativeness[Title/Abstract]))) AND ((((((("Associated Factors"[Title/Abstract]) OR (Determinants[Title/Abstract])) OR ("Epidemiologic factors"[Title/Abstract])) OR (Predictors[Title/Abstract])) OR ("Risk factors"[Title/Abstract])) OR ("Contributing Factors"[Title/Abstract])) OR (Correlates[Title/Abstract]))) AND (((((((Adult[Title/Abstract]) OR ("adult"[MeSH Terms])) OR ("Post-adolescent"[Title/Abstract])) OR ("Young adult"[Title/Abstract])) OR ("Middle-aged"[Title/Abstract])) OR (Elderly[Title/Abstract])) OR ("Adult patients"[Title/Abstract]))) AND (((("Intensive Care Unit"[Title/Abstract]) OR ("Intensive care"[Title/Abstract])) OR ("Critical care"[Title/Abstract])) OR (ICU[Title/Abstract])) **(n=108)** |
| **Google Scholar** | ("Agitation" OR "psychomotor agitation") AND ("Adult ICU" OR "Intensive Care Unit" OR "Critically Ill Patients") AND ("Incidence" OR "Prevalence" OR "Magnitude") AND ("Associated Factors" OR "Predictors" OR "Risk factors") **(N=18,100)** |
| **Science Direct** | (Agitation ) AND ("intensive care unit" OR ICU OR "critical care") AND (incidence ) AND ("associated factors" OR "risk factors" OR predictors) **(N=6,178)** |
| **Cochrane Liberary** | ("Agitation" OR "psychomotor agitation") AND ("Adult ICU" OR "Intensive Care Unit" OR "Critically Ill Patients") AND ("Incidence" OR "Prevalence" OR "Magnitude") AND ("Associated Factors" OR "Predictors" OR "Risk factors") **(n=1)** |
| **Total** | **24,387** |
